# Supplementary material for: Dendrogenin A Enhances Anti-Leukemic Effect of Anthracycline in Acute Myeloid Leukemia
Source: Cancers (Basel). 2020 Oct 12;12(10):2933. doi: 10.3390/cancers12102933 (PMC7601603; doi:10.3390/cancers12102933)
Supplement: Supplementary file 1 [file cancers-12-02933-s001.pdf]

Supplementary Materials

Dendrogenin a Enhances Anti-Leukemic Effect of Anthracycline in Acute Myeloid Leukemia

Pierre-Luc Mouchel, Nizar Serhan, Rémy Betous, Thomas Farge, Estelle Saland, Philippede Medina, Jean-Sébastien Hoffmann, Jean-Emmanuel Sarry, Sandrine Silvente-Poirot, Marc Poirot and Christian Récher

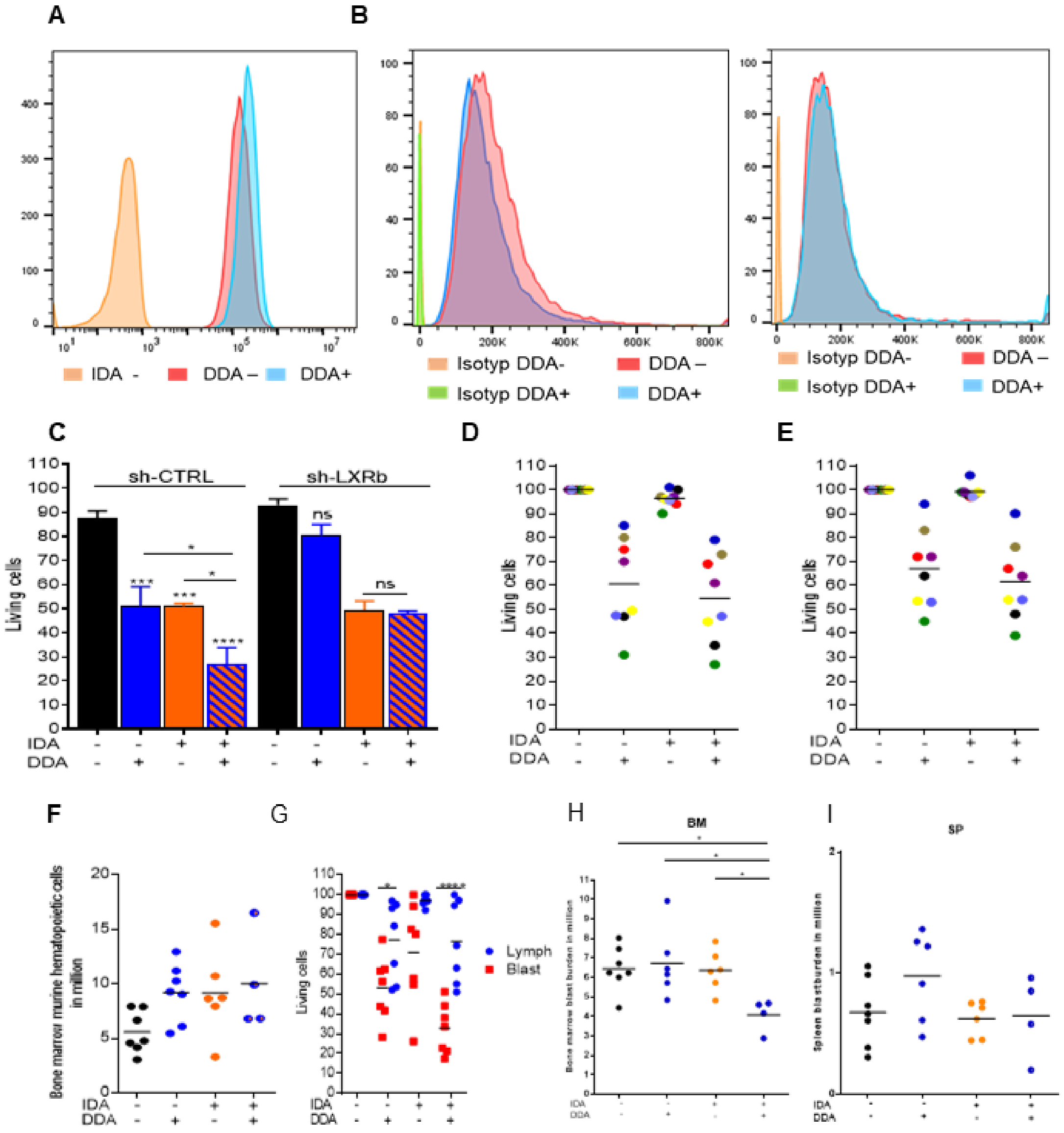

Figure S1. Combined DDA and daunorubicin synergistically induce the death of AML cells. (**A**) Intra-cellular daunorubicin measurement by flow cytometry on KG1a cells after 24 h pretreatment with DDA (2.5) or vehicle and treated 30 min with daunorubicin or vehicle. Data were represented as fluorescence intensity relative, in annexin-V-/7AAD-cells. (**B**) PgP membrane expression measurement by flow cytometry on KG1-shCTRL or KG1-sShLXRβ cells after 24 h treatment with DDA (2.5) or vehicle. Data were represented as fluorescence intensity relative, in annexin-V-/7AAD-cells. (**C**) Cell death measurement by Trypan Bleu exclusion test on shCTRL-and shLXRβ-expressing KG1 cells treated for 48 h with DDA (5 µM), IDA (50 nM) or both DDA and IDA (5 µM/50 nM). Bars are mean ± SD of 3 independent experiments. (**D**) PBMC from healthy controls (*n*

= 8) samples were treated with DDA (2.5 μM) or IDA (10 nM) or both DDA and IDA (2.5/10 nM) or vehicle for 48 h. Cell death was assessed in all hematopoietic cells (CD45+) using annexinV/7AAD staining. Data were represented as % of survival corresponding to annexin-V-/7AAD-cells relative tototal cells. (**E**) PBMCs from healthy controls (*n* = 8) samples were treated with DDA (2.5 μM) or IDA (10 nM) or both DDA and 20IDA (2.5/10 nM) or vehicle for 48 h. Cell death was assessed in T-and B-lymphocytes (CD45+CD3+ or CDCD45+CD19+) using annexinV/7AAD staining. Data were represented as % of survival corresponding to annexin-V-/7AAD-cells relative to total cells. (**F**) Hematopoietic cell content in bone marrow and spleen was measured by flow cytometry using human anti-CD45, anti-CD45.1 and human anti-CD33 antibodies. (**G**) In 7 blood samples from leukemic patients, the response of AML cells and normal lymphocytes treated 48 h with vehicle, DDA, IDA or both IDA and DDA were analyzed. Data were represented as % of survival corresponding to annexin-V-/7AAD-cells relative to total cells in lymphocytes (blue) or AML blasts (red). (**H**) Bone marrow cell tumor burden of MOLM14 orthotopically xenografted NSG mice were treated with DDA (20 mg/kg/day by i.p injection) or IDA (0.15 mg/kg/day every two days for 5 days by i.v injection) or both DDA and IDA or vehicle control. MOLM-14 leukemic cell burden in bone marrow and spleen was measured by flow cytometry using human anti-CD45, anti-CD45.1 and human anti-CD33 antibodies. (**I**) Spleen cell tumor burden of MOLM14 orthotopically xenografted NSG mice were treated with DDA (20 mg/kg/day by i.p injection) or IDA (0.15 mg/kg/day every two days for 5 days by i.v injection) or both DDA and IDA or vehicle control. MOLM-14 leukemic cell burden in bone marrow and spleen was measured by flow cytometry using human anti-CD45, anti-CD45.1 and human anti-CD33 antibodies. * $p < 0.05$, ** $p < 0.01$ and *** $p < 0.001$.

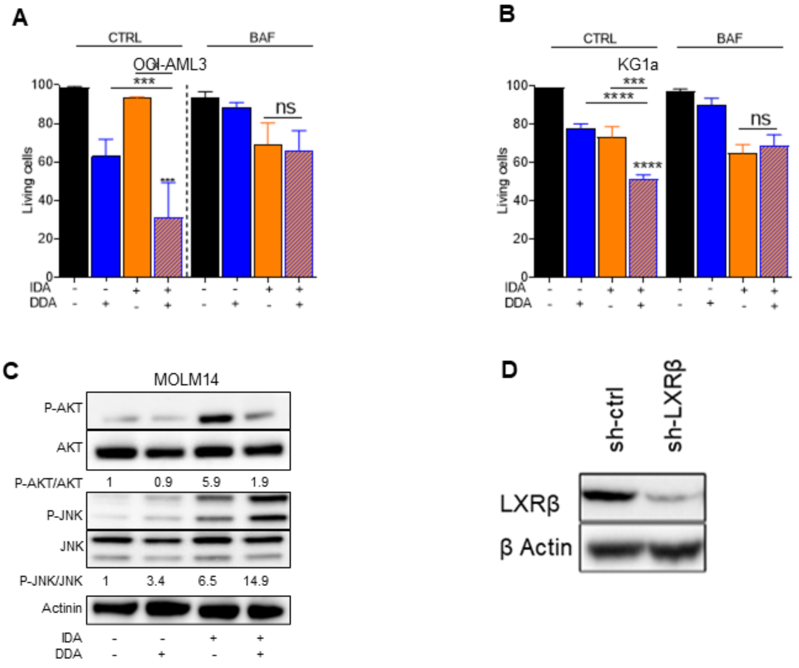

Figure S2. Implication of autophagy after DDA-IDA treatment. (**A**–**B**) Cell death measurement by flow cytometry after autophagy inhibition on OCI-AM3 and KG1a cells treated for 48 h with DDA, IDA or both DDA and IDA in the presence or absence of bafilomycin A1. Data were represented as % of survival corresponding to annexin-V-/7AAD-cells. Bars are mean ± SD of 3 independent experiments. Cell death measurement by Trypan Blue exclusion test after autophagy inhibition on MOLM-14 cells treated for 48 h with DDA, IDA or both DDA and IDA in the presence or absence of bafilomycin A1. Bars are mean ± SD of 3 independent experiments. (**C**) Western blot analysis of the expression of p-AKT, AKT, JNK and p-JNK in MOLM-14 cells treated 5 h with vehicle, DDA, IDA or both IDA and DDA. (**D**) Immunoblot of LXRβ expression in KG1 cells stably transfected with control shRNA (sh-CTRL) or with shRNA against LXRβ (Sh-LXRβ). * $p < 0.05$, ** $p < 0.01$ and *** $p < 0.001$.

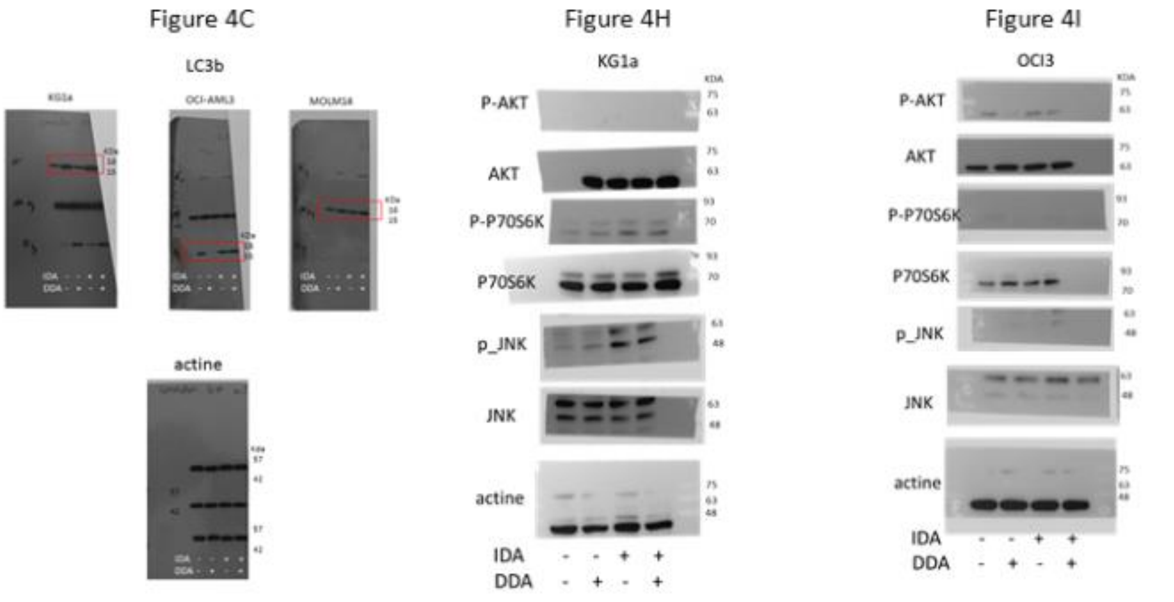

Figure S3. Uncropped Western Blot from Figure 4.

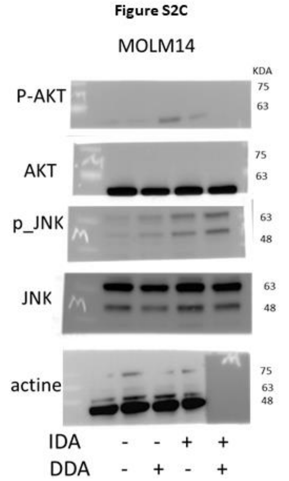

Uncropped Western Blot from Figure S2.

Table S1. Values of combination index presented in Figure 1.

Cell Line	Affected Fraction	Combination Index	Cell Line	Affected Fraction	Combination Index	Cell Line	Affected Fraction	Combination Index
KG1	0.1	0.63	MV4-11	0.1	0.4714	KG1a	0.1	0.214
KG1	0.2	0.6818	MV4-11	0.2	0.5	KG1a	0.2	0.178
KG1	0.3	0.697	MV4-11	0.3	0.5143	KG1a	0.3	0.15
KG1	0.4	0.7121	MV4-11	0.4	0.5429	KG1a	0.4	0.125
KG1	0.5	0.7424	MV4-11	0.5	0.5571	KG1a	0.5	0.098
KG1	0.6	0.7576	MV4-11	0.6	0.5786	KG1a	0.6	0.071
KG1	0.7	0.7727	MV4-11	0.7	0.6	KG1a	0.7	0.0625
KG1	0.8	0.7879	MV4-11	0.8	0.621	KG1a	0.8	0.0536
KG1	0.9	0.8788	MV4-11	0.9	0.6643	KG1a	0.9	0.0447

© 2020 by the authors. Licensee MDPI, Basel, Switzerland. This article is an open access article distributed under the terms and conditions of the Creative Commons Attribution (CC BY) license (http://creativecommons.org/licenses/by/4.0/).
